# Editorial: Teaching and learning Chinese as a foreign or second language: the educational psychology perspective

**DOI:** 10.3389/fpsyg.2024.1333836

**Published:** 2024-02-12

**Authors:** Yang Frank Gong, Chun Lai

**Affiliations:** ^1^Chinese Education Programme, Faculty of Education, University of Macau, Zhuhai, Macau SAR, China; ^2^Language and Literacy Education Unit, Faculty of Education, The University of Hong Kong, Pokfulam, Hong Kong SAR, China

**Keywords:** educational psychology, Chinese as a second/foreign language (CSL/CFL), CSL/CFL learners, language acquisition, teacher development

## 1 Introduction

Chinese, as a globally significant language widely used both within and outside of China, has witnessed a remarkable increase in the number of learners worldwide (Gong et al., 2018, 2020). By the end of 2021, the number of people outside China learning Chinese as a second/foreign language (CSL/CFL) had exceeded 25 million, with over 200 million individuals learning and using the language (Ministry of Education, 2023). Understanding how CSL/CFL learners acquire and develop the language and helping them overcome learning difficulties and challenges through effective strategies are hence essential. Educational psychology provides useful lenses to shed light on these issues. There is a growing number of studies that adopt psychological perspectives to investigate CSL/CFL learning and teaching across the world. These studies have explored the psychological issues and mechanisms behind learners' acquisition of Chinese characters, phonology, vocabulary, and grammar, adopted a cultural psychological lens to unravel the role of Chinese cultural contexts in influencing learners' Chinese language acquisition and development, and examined the motivational sources that drive student learning and the factors that contribute to teachers' professional development (Ma et al., 2017; Gong et al., 2020). To enrich this body of literature, this Frontiers Research Topic collects a series of studies that examine various aspects of CSL/CFL teaching and learning from an educational psychology perspective.

## 2 Research on CSL/CFL teaching and learning from an educational psychology perspective

Psychological perspectives provide valuable insights into second language acquisition. Studies adopting the educational psychology perspective have shed light on various psychological issues, such as language aptitude, motivation, learning strategies, and identity (Dörnyei, 2014; An et al., 2024), that are critical to successful language learning, and conceptualized and tested models of psychological mechanisms behind the development of language learning outcomes, such as phonology, vocabulary, and grammar/sequence rules (Wen et al., 2017). These studies have also revealed how learners' cognitive constraints, strategy choices, and learning experience construction influence their language proficiency (Shen, 2013; Liu et al., 2017; Szyszka, 2017). By understanding these factors, educators and researchers can better facilitate learners in their language learning and development journey.

However, most of these studies have been conducted on the acquisition of anglophone languages. However, the characteristics of the languages and culturally-shaped learning experiences influence the neuro-psychological mechanisms activated for language processing and acquisition (e.g., Zhang et al., 2023). Scholars have also underscored cultural specificity in various psychological constructs, such as motivation (e.g., Wang et al., 2020), and its impact on learning. Therefore, further research from an educational psychology perspective is necessary to enhance our understanding of CSL/CFL development. Such research can contribute to expanding our knowledge of psychological issues in language acquisition more broadly by providing language- and culture-specific insights. Moreover, the development of technology and associated shift or expansion of language learning contexts also demands research into psychological issues in these new learning contexts. Take research on engagement, anxiety, and motivation as an example. The effects of these psychological factors on language proficiency have been widely acknowledged in the traditional offline classroom setting (Basith et al., 2019; Gong et al., 2020; Hiver et al., 2021). However, the COVID-19 pandemic has necessitated a shift to online instruction in language classes, which creates a different research context for CSL/CFL education (Chen, 2021). In light of this shift, collaborative efforts are needed to attain a more comprehensive understanding of CSL/CFL learners' anxiety, motivation, and ability to maintain engagement and learning stickiness in online learning environments. In addition, CSL/CFL teachers not only face challenges regarding facilitating learners' Chinese acquisition and development but also the reformation of their professional identity. Teacher identity, which is influenced by a global mindset and intercultural competence, needs to be renegotiated in diverse social-cultural contexts (Gong et al., 2022). Thus, further studies are required to explore how CSL/CFL teachers can effectively navigate the socio-cultural landscape, foster meaningful interactions in social networks, and enhance their professional development and CSL/CFL teaching quality.

## 3 This Research Topic

We received 60 abstracts and 59 manuscript submissions in total, and there are 23 articles finally published through rigorous peer review. In other words, the overall acceptance rate of this Research Topic is 19.3%, which indicates it is one of the most high-quality Frontiers Research Topic. The published articles featured in this Research Topic cover a wide range of topics, being classified into three main categories based on their research themes: (1) psychological factors in CSL/CFL acquisition, (2) learners' anxiety, motivation, and strategy related to CSL/CFL learning, and (3) teacher training and teachers' agency and identity concerned with CSL/CFL teaching. One common thread among all the papers is the recognition of the significance of integrating the psychology perspective into CSL/CFL education research.

### 3.1 Psychological factors in CSL/CFL language acquisition (11 articles)

The 11 articles in this category focus on the influence of psychological factors on CSL/CFL acquisition of linguistic aspects and skills, with a particular emphasis on learners' awareness and strategies in the acquisition process as well as teachers' instructional strategies.

Regarding lexicology acquisition difficulties, researchers tend to concentrate on learners' lexical reasoning abilities in their CSL/CFL learning. Zhang H. et al. analyzed a vocabulary assessment and a lexical inference task and revealed that vocabulary knowledge contributed to Chinese lexical reasoning skills of 419 heritage learners at different proficiency levels. The influence of heritage background on morphological awareness and lexical inference was stronger than that of the Chinese language proficiency level. Considering the early exposure of heritage learners to spoken language, it is recommended that they can enhance their lexical inference abilities by explicit instruction on the segmentation bimorphemic and multi-morphemic words. Jin et al. also paid attention to Chinese heritage language speakers. Implementing an online language acceptability judgment task, they examined learners' ability to accurately process and understand different types of nominal expressions in Chinese in actual time that were regulated by syntax-semantics and syntax-discourse interfaces. This study suggested that language-external interface properties were not necessarily destined for prolonged difficulties for these learners. Along this research line, Zheng et al. pointed out that the Chinese proficiency level impacted the accuracy and speed of processing Chinese idioms vs. non-idiomatic formulaic sequences, and in particular, learners' ability to resolve difficulties in the use of idioms improved with the level of Chinese language proficiency. They also suggested some classroom teaching approaches to improve students' ability to utilize idioms according to learners' cognitive characteristics. In terms of Chinese semantics, Fang and Xu compared online sentence-picture matching to offline translation tasks between 31 English learners and 29 native Mandarin Chinese speakers and demonstrated a prototypical effect, indicating that CSL learners found it easier to comprehend the associations between aspect markers and specific predicate types. A study by Liu and Ning on 20 Cantonese native speakers, 18 Cantonese-dominants, and 18 Urdu-dominants showed that Urdu-dominant speakers did not pay as much attention to tones and did not exhibit greater perceptual flexibility than Cantonese-dominant bilinguals when processing Cantonese stimuli. Studies pertinent to language skills have emphasized the importance of reading comprehension, writing, and pragmatics skills in Chinese language learning. Researchers investigated CSL/CFL learners' language skills in terms of character, vocabulary, and discourse, incorporating both their input and output. Liao et al. conducted two assessments focusing on Chinese character reading and reading comprehension with secondary school students in Hong Kong SAR and found that both lexical orthographic choice itself and lexical orthographic choice in context played a mediating role in reading comprehension, but the latter was more crucial. Concerning vocabulary use, Zhang L. et al. focused on connective errors in CSL learners' writing. By ranking the most common error types, they found that intralingual transfer significantly led to such errors and suggested that Chinese language teachers could use appropriate teaching strategies to help students reduce errors. Moving to individual differences and self-regulation of CSL/CFL learners at mainland Chinese universities, studies conducted by Lv et al. and by Zhang J. et al. focused on students' discourse output abilities and beliefs. They revealed an association between perceived communicative competence and increased pragmatic comprehension. However, they found there was no significant relationship between willingness to communicate in a second language (L2) and self-perceived communicative competence. At the same time, Zhang J. et al. reported that students' beliefs about corrective feedback were associated with their language accuracy. They suggested that language teaching methods derived from research on EFL/ESL learners' corrective feedback were similarly applicable to CSL/CFL learning.

Two studies explored form-focused instruction (FFI). In Chen and Li's study, they found that both focus-on-forms (FonFs) and focus-on-form (FonF) could enhance CSL learners' verbal communication skills. FonFs was more effective in improving fluency for students with low language competence, whereas FonF was more effective in increasing accuracy for students with high language proficiency. Regarding Chinese writing, Zhou and Lü found that FFI on thematic chains had a positive and long-lasting impact on participants' syntactic complexity. The effectiveness and durability of FFI were related to the intensity of instruction and the type of feedback given. Therefore, FFIs played a crucial role in teaching students to improve their speaking and writing skills.

Based on the above-mentioned discussions of psychological factors influencing CSL/CFL education, it is evident that integrating psychological perspectives and approaches into language education can effectively understand learners' learning experience and outcomes.

### 3.2 Learners' anxiety, motivation, and strategy (six articles)

Previous studies have provided valuable insights into CSL/CFL learners' anxiety, motivation, and strategy, with a focus on context-specific learning dilemmas and solutions. In a systematic review study, Yao et al. reported that international researchers showed a growing interest in Research Topics related to CSL/CFL learning anxiety compared to scholars in mainland China. The latter had limited practice of qualitative approaches, such as interviews and class observations, indicating the need for mainland Chinese researchers to keep abreast of the latest theories and methodologies in the field.

Researchers have paid attention to examining the psychological attributes associated with CSL/CFL learners' learning process in the online instructional context during the COVID-19 pandemic. Xu et al. focused on online Chinese language learners and analyzed their self-reports and vocabulary knowledge. The findings showed that anxiety had a greater impact on student performance than motivation and learning strategies. However, all three factors were only correlated with self-assessed Chinese language proficiency, not performance in Chinese vocabulary assessment. This study contributed to the comprehension of the link between L2 learning performance and individual discrepancy in online learning contexts. Lin, Gong et al. conducted a study with 378 international students who were enrolled in online Chinese language classes, to investigate their profiles of self-regulated learning (SRL) using a person-centered approach. The study employed a learning motivation assessment that encompassed students' learning anxiety, goal orientation, task value, and learning strategy scale. The findings of this study provided evidence in support of the situational constraints on SRL. They emphasized that L2 teachers should implement more effective classroom practices to facilitate students' SRL according to the discrepancy.

The social context has a significant influence on learners' engagement and strategy use in language learning. Chen found that engagement in online Chinese classes was influenced by learning expectations, academic and social environments, and educational technology readiness. In these factors, social environments were observed as a significant predictor, and thus teachers were advised to have more social interaction online with students, which is usually overlooked and unnecessary in offline classes. The social contextual nature together with learners' agency were found to impact learners' strategy choices and use in the study of Li et al.. Teachers, as a mediating role, were suggested to fully understand students' learning strategies and integrate online Chinese language learning resources to create and adapt appropriate social-cultural experiences for students. In line with the role of social-cultural contexts, Lin, Lam et al. used a reading assessment and questionnaire to analyze learners' motivation and learning strategies and their comprehension skills. Learning strategies employed by CSL/CFL students were impacted by the socio-cultural context and learning motivation strategies had both indirect and direct effects on comprehension performance. This study could guide the teachers in selecting the group of developmental strategies to instruct students appropriately to respond to specific reading tasks.

The above-mentioned findings suggest that environmental constraints in online learning impact CSL/CFL learners' anxiety and engagement and individual learners can adapt to contextual challenges by employing diverse learning strategies under the instruction.

### 3.3 Teacher training, teacher agency and identity, and teacher development (six articles)

Researchers explored CSL/CFL teacher training, teacher agency and identity, and teacher professional development from a psychological perspective, focusing on their interaction with the socio-cultural context. Gong et al. examined preservice CSL teachers' understanding of culture and intercultural teaching and found their teaching objectives were mainly attitude-oriented, lacking the orientation toward knowledge and skills. This research calls for a more innovative curriculum to train preservice CSL teachers. Gu et al. explored the agency of Chinese language teachers in constructing their professional identity and compared the experiences of native and non-native Chinese language teachers with regard to various factors related to professional motivation. The study discovered noteworthy disparities in the perceived extrinsic value and social influence between the two participant groups, with only minor variations observed in terms of cultural, intrinsic, and altruistic value as well as future career development choices. These findings highlight the unique nature of professional motivation among L2 teachers. Chen et al. investigated the teachers' identity in terms of interacting with students. The study showed teachers' intergroup uniqueness (identity attributes) remained unchanged although they faced complicated cultural backgrounds of students. It is agreed that the identity construction of CFL teachers was influenced by their self-identification and social integration. Yang and Han conducted a study using retrospective narrative inquiry to document the 10-year experiences of a CFL teacher and indicated that teacher agency influenced the implementation of identity-oriented instructional practices. Similar findings are also reported in the study of Han and Ji, which examined the interaction between three pre-service CFL teachers and their new sociocultural context from a Chinese-Australian bilateral provincial master's degree program. CSL teachers in Australia faced challenges in professional identity development, which were affected by self-identification and their interaction with others in the community. Such difficulties could lead to uncertainty and confusion and hinder the teacher's ability to perform their duties effectively. In addition, Yang et al. revealed that CSL teachers' personal networks played a significant role in their agency enactment. These personal networks guided their values, provided emotional and academic support, and helped them become effective agents in the face of diverse educational challenges. In this regard, networking is a crucial means for teachers to enhance their academic and professional competency and broaden their learning opportunities throughout their careers. The findings of these studies highlight the importance of adopting the psychological perspective in research on CFL/CSL teachers' motivation and identity reformation in diverse cultural contexts.

## 4 Future directions: where shall we go?

This special edition draws attention to the understanding and examination of CSL/CFL teaching and learning from an educational psychology perspective. Overall, the 23 articles included in this Research Topic address various research issues with different foci. Their common aim is to explain and analyze the psychological factors or processes related to students' second/foreign language acquisition, learning strategies as well as teachers' instructional practices and professional development. In this regard, the findings from this special edition provides an opportunity for language education researchers and practitioners to reconsider their understanding of second/foreign language education, which has been primarily based on inquiries into commonly taught English (Gao and Zheng, 2019; Gong et al., 2020). To support the growth of multilingual and intercultural education, we hope that these publications can inspire education stakeholders to reflect on the application of psychology into authentic CSL/CFL teaching and learning, and encourage them to make conceptual or theoretical contributions to second/foreign language education. At the same time, researchers need to consider using psychological perspectives to address issues and promote the quality of CSL/CFL teaching and learning in different educational contexts. In other words, CSL/CFL research should be encouraged and expanded to examine a broader range of Chinese language learners, both in and outside of China, who learn and use Chinese in diverse contexts. For instance, researchers need to pay more attention to the inheritance and maintenance of Chinese as a heritage language (CHL) and CHL learners' interaction with the social, cultural, and political context of Chinese learning (Mu, 2016).

## Author contributions

YG: Conceptualization, Supervision, Writing – original draft, Writing – review & editing. CL: Conceptualization, Writing – review & editing.
